# Efficacy of Group Exercise–Based Cancer Rehabilitation Delivered via Telehealth (TeleCaRe): Protocol for a Randomized Controlled Trial

**DOI:** 10.2196/38553

**Published:** 2022-07-18

**Authors:** Amy M Dennett, Katherine E Harding, Casey L Peiris, Nora Shields, Christian Barton, Lauren Lynch, Phillip Parente, David Lim, Nicholas F Taylor

**Affiliations:** 1 Allied Health Clinical Research Office Eastern Health Box Hill Australia; 2 School of Allied Health, Human Services and Sport La Trobe University Bundoora Australia; 3 Community Health Eastern Health Healesville Australia; 4 Department of Cancer Services Eastern Health Box Hill Australia; 5 Eastern Health Clinical School Monash University Clayton Australia; 6 School of Health Sciences Western Sydney University Campbelltown Australia

**Keywords:** telehealth, exercise, telerehabilitation, physical activity, supportive care, cancer

## Abstract

**Background:**

Access to rehabilitation to support cancer survivors to exercise is poor. Group exercise–based rehabilitation may be delivered remotely, but no trials have currently evaluated their efficacy.

**Objective:**

We aimed to evaluate the efficacy of a group exercise–based cancer rehabilitation program delivered via telehealth compared to usual care for improving the quality of life of cancer survivors.

**Methods:**

A parallel, assessor-blinded, pragmatic randomized controlled trial with embedded cost and qualitative analysis will be completed. In total, 116 cancer survivors will be recruited from a metropolitan health network in Melbourne, Victoria, Australia. The experimental group will attend an 8-week, twice-weekly, 60-minute exercise group session supervised via videoconferencing supplemented by a web-based home exercise program and information portal. The comparison group will receive usual care including standardized exercise advice and written information. Assessments will be completed at weeks 0 (baseline), 9 (post intervention), and 26 (follow-up). The primary outcome will be health-related quality of life measured using the European Organisation for Research and Treatment of Cancer Quality of Life Questionnaire at week 9. Secondary measures include walking capacity (6-minute walk test), physical activity (activPAL accelerometer), self-efficacy (Health Action Process Approach Questionnaire), and adverse events. Health service data including hospital length of stay, hospital readmissions, and emergency department presentations will be recorded. Semistructured interviews will be completed within an interpretive description framework to explore the patient experience. The primary outcome will be analyzed using linear mixed effects models. A cost-effectiveness analysis will also be performed.

**Results:**

The trial commenced in April 2022. As of June 2022, we enrolled 14 participants.

**Conclusions:**

This trial will inform the future implementation of cancer rehabilitation by providing important data about efficacy, safety, cost, and patient experience.

**Trial Registration:**

Australian New Zealand Clinical Trials Registry ACTRN12621001417875; https://tinyurl.com/yc5crwtr

**International Registered Report Identifier (IRRID):**

PRR1-10.2196/38553

## Introduction

There are clear clinical practice recommendations to integrate exercise-based rehabilitation into cancer care [1,2]. However, access to exercise-based cancer rehabilitation is poor with just 1 in 200 cancer survivors able to access appropriate support [3]. This is a major concern given that exercise-based rehabilitation mitigates negative side effects of cancer treatment such as fatigue and depression and improves physical function and quality of life [4]. High levels of exercise after diagnosis can reduce the risk of developing comorbidities such as cardiovascular disease and is associated with a reduction in cancer-related death [5] and cancer recurrence [6]. Exercise-based cancer rehabilitation can facilitate the return to normalcy and establish positive lifestyle changes to prevent long-term morbidity [7].

In-person interventions are the standard for delivering exercise-based cancer rehabilitation. While effective at improving patient outcomes, in-person exercise-based cancer rehabilitation programs delivered in clinical settings often have poor adherence and attendance [8,9] owing to patient-related issues such as fatigue [10] and managing competing medical demands [11]. Other issues that can limit access and diminish the effectiveness of exercise-based cancer rehabilitation include logistical problems such as cost, parking, and location [3,11,12].

Telehealth may overcome barriers related to in-person care delivery. Telehealth uses technologies such as videoconferencing, telephone, and mobile apps for diagnosis, treatment, and prevention of disease [13]. An advantage of telehealth is convenience, and it has been described by cancer survivors as minimizing the treatment burden [14]. Rehabilitation delivered by telehealth (hereafter referred to as “telerehabilitation”) can be used to implement the key elements of cancer rehabilitation including exercise demonstration, instruction, observation, and information provision. The feasibility of telerehabilitation has been established in a cancer context. Telerehabilitation interventions are safe, have good adherence, and provide a positive patient experience among cancer survivors [15,16]. Individual telerehabilitation improves physical activity levels and quality of life of cancer survivors when compared to usual care without exercise [17-19]. A phone-based telerehabilitation intervention compared with usual care focused on pain reduction, improved mobility, reduced pain, and hospital length of stay in people with advanced cancer [20]. However, despite the broad variety of telehealth technologies that are available, most trials investigating exercise telehealth interventions for people with cancer have used simple, individual telephone interventions [21-24].

Current telehealth approaches to exercise rehabilitation are limited in their ability to replicate traditional cancer rehabilitation. To date, no trials have evaluated the effectiveness of web-based group exercise for people with cancer [15,23]. Group exercise interventions may be superior to other exercise interventions [25] and are the most common way to deliver cancer rehabilitation in health services [3]. Groups provide a positive environment for exercise for cancer survivors [7] and allow opportunities for peer support, modeling, and feedback [26]. Groups are also an efficient way to deliver exercise that may facilitate access and be less resource-intensive on health services. There is potential for exercise groups to be delivered via telehealth as videoconferencing technology can enable the supervision of multiple participants. Since the COVID-19 pandemic, there has been a surge in the use of telehealth such as for the provision of group exercise [16,27]. Therefore, robust trials of group telerehabilitation including exercise for cancer survivors are required to determine their efficacy and effectiveness.

The primary aim of this pragmatic randomized controlled trial is to evaluate the efficacy of an exercise-based telerehabilitation program compared to usual care for improving the quality of life of cancer survivors. Secondary aims are to compare the effects of cancer telerehabilitation on walking capacity, physical activity levels, self-efficacy, and adverse events. We will also determine the costs associated with telerehabilitation and explore, in depth, the experience of cancer survivors completing cancer telerehabilitation.

## Methods

### Study Design

**Figure 1:**
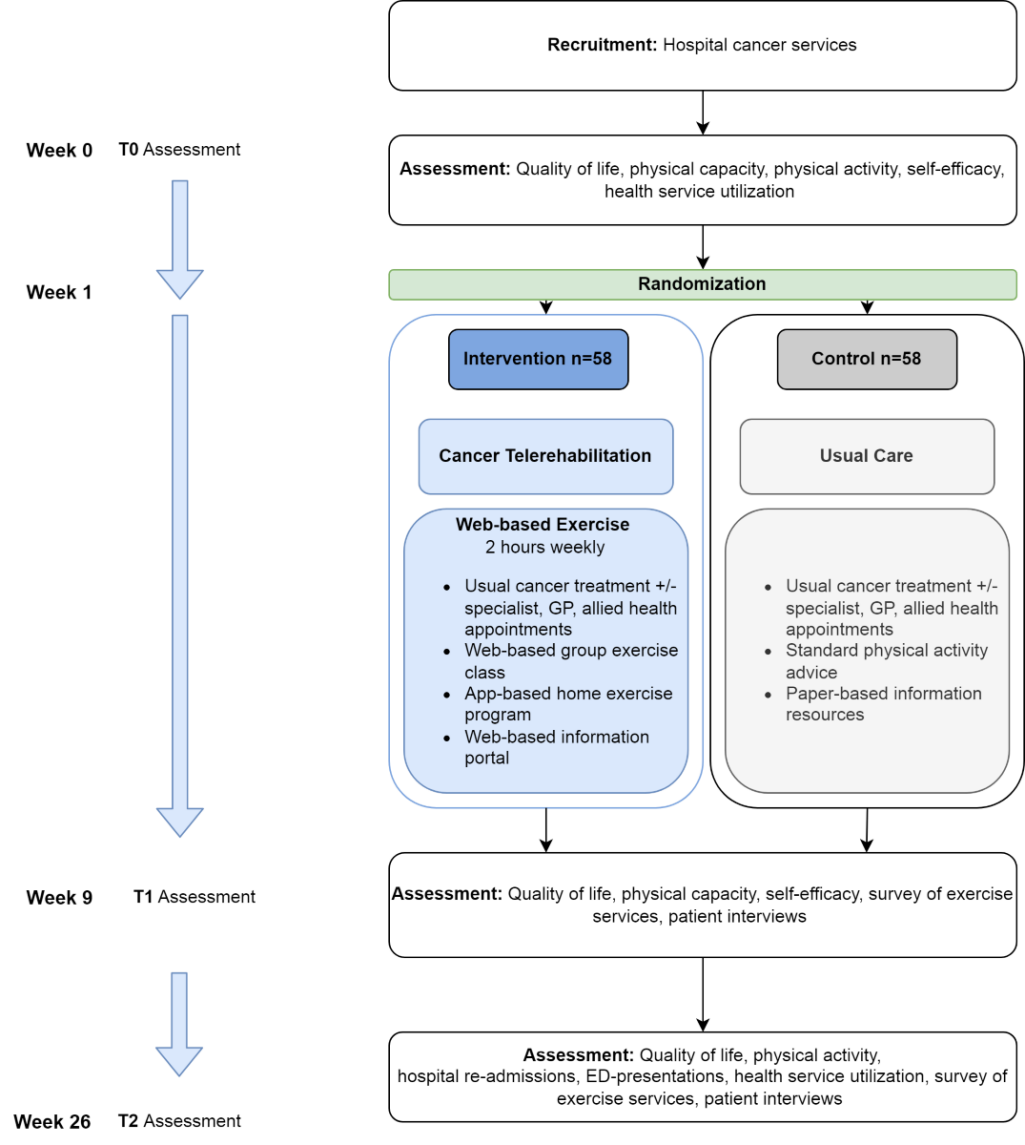
CONSORT (Consolidated Standards of Reporting Trials) diagram for the TeleCaRe trial. ED: emergency department; GP: general practitioner.

We will complete a prospective, parallel, assessor-blinded, pragmatic randomized controlled trial comparing the efficacy and cost-effectiveness of 8 weeks of group exercise–based cancer telerehabilitation to usual care (see Figure 1 for the study flowchart). Participants will be assessed at weeks 0 (baseline), 9 (post intervention), and 26 (follow-up) (Multimedia Appendix 1). Quantitative trial outcomes will be reported in accordance with the CONSORT (Consolidated Standards of Reporting Trials) statement [28], qualitative outcomes reported with consolidated criteria for reporting qualitative research [29] and health economic analysis reported with the Consolidated Health Economic Evaluation Reporting Standards [30]. The qualitative component of the study will be conducted using an interpretivist paradigm, which recognizes that multiple realities exist and this is affected by context [31].

### Randomization Procedures

Eligible participants who have completed baseline measurements will be randomly allocated to the telerehabilitation group or usual care control group using a concealed method in accordance with a web-based computer-generated randomization program using permuted blocks of 4, 6, and 8 participants. Allocations will be prepared prior to trial commencement by an independent researcher with no role in participant recruitment, trial administration of intervention delivery, or assessments. The trial coordinator will allocate participants after baseline assessment by contacting the independent researcher via email for random group allocation.

### Setting

The trial will be conducted in a large public health network in Melbourne, Victoria, Australia, which services approximately 3000 cancer survivors annually. Participants will be recruited from cancer services at 3 metropolitan sites within this single health network.

### Ethics Approval

The TeleCaRe trial has been approved by the Eastern Health and La Trobe University Human Research Ethics Committees (E21-012-74698) and is funded by the Victorian Cancer Agency.

### Patient Selection and Consent

Eligible participants will be identified by any member of the cancer services clinical team at the health network (eg, oncologists, nursing staff, and physiotherapists). Potential participants will be advised about the trial by clinic staff verbally or through flyers. If a patient consents, he/she will be contacted by a member of the research team who will provide details of the trial and arrange an outpatient appointment at home or at the clinical site to provide an opportunity for questions to be clarified and to provide written informed consent.

### Inclusion and Exclusion Criteria

Participants will be eligible if they are aged 18 years and over, have a cancer diagnosis and are receiving cancer treatment (palliative or curative intent) or are within 12 months of completing adjuvant therapy (except for long term oral hormonal therapies), are functioning independently in the community (Australian Karnofsky Performance Status [AKPS] score≥60), are at a low risk of falls (Falls Risk for Older People in the Community score<4), are able to speak conversational English so participants can engage effectively in videoconferencing, have access to and be willing to use the internet, and be able to give written informed consent.

Participants will be excluded if they are medically unfit to participate in exercise as determined by a physiotherapist or medical practitioner on the basis of published recommendations [32], are residing in residential care or are an inpatient, or have cognitive impairment precluding the ability to provide written informed consent as assessed by their treating clinician.

### Intervention

All participants will receive their usual medical care, which may include adjuvant, neoadjuvant, or palliative treatment, specialist, nursing and other health outpatient appointments (eg, to see a physiotherapist) and visits to their general practitioner.

As part of the trial, all participants will be provided with written educational materials relating to different aspects of cancer recovery (eg, exercise, nutrition, and fatigue) via standardized print or digital material readily available from the hospital.

Usual care in the community involves very little exercise support in relation to exercise. As part of the trial, usual care also includes standardized verbal and written advice to complete physical activity in line with current recommendations (aim for 3 times weekly exercise for 30 minutes, including twice weekly strength training) [4]. All participants will also have the opportunity to discuss their ongoing rehabilitation needs at the end of the 8-week intervention period. They will be provided written information for referral to appropriate local services for ongoing support if required in line with usual practice at the health service.

### Experimental Group: Exercise-Based Cancer Telerehabilitation

In addition to usual care, participants randomized to the experimental group (telerehabilitation) will receive a 60-minute group exercise delivered by a physiotherapist via videoconferencing (Zoom) twice-weekly for 8 weeks. Exercise will comprise cardiovascular and resistance training guided by published recommendations [4]. Exercise sessions will be individually tailored and include the use of free weights, resistance bands, body weight, and functional activities. Supervised aerobics (eg, marching in place and side-stepping) will comprise the cardiovascular component. Participants will be provided with an exercise band, and the exercise program will be supplemented by participants’ own exercise equipment or household items. The therapist will choose an exercise variation (eg, bicep curl using weights or with exercise band) based on the equipment available to the participant. Various upper- and lower-body stretches and balance exercises will be incorporated as required. Exercise intensity will be monitored during the exercise class using a Fitbit device (Fitbit Inspire) and modified Borg scale. The Fitbit will also be used for participants to self-monitor their physical activity levels throughout the 8-week intervention. A home exercise program will supplement exercise-based telerehabilitation sessions and be delivered via a web application (Physitrack). The home exercise program will encourage one additional 30-minute aerobic exercise session per week (eg, walking) during the intervention period and will be updated at the end of the rehabilitation program to encourage participants to conduct twice-weekly strength training and 3 times weekly aerobic training after the 8-week intervention period. Participants will also be provided education materials relating to different aspects of cancer recovery (eg, nutrition, emotions, and fatigue) via a web-based information portal (iLearn, Totara Learning Solutions, Wellington, New Zealand) (Table 1).

Physiotherapists conducting assessments and providing the exercise intervention will be trained in exercise rehabilitation for cancer survivors. They will participate in a 1-day web-based training session about cancer care and complete a self-directed web-based module about cancer-related fatigue management. They will also participate in 2 interactive 2-hour in-person workshops about exercise prescription for cancer survivors. They will also have access to a website [33] that provides education for clinicians providing exercise-based cancer rehabilitation. Physiotherapists will receive monthly clinical supervision in line with health service policy and regular mentoring with senior research staff. The fidelity of the intervention will be monitored by recording the content of exercise sessions using logbooks, including the number and duration of completed sessions. Participants in both groups will also be asked whether they received any exercise-based intervention outside of the trial at the 9-week assessment and 4-month follow-up.

**Table 1:** Intervention and comparison group descriptions using the template for description and replication checklist (TIDier).

			Experimental group		Comparison group
Brief name			Group exercise–based cancer telerehabilitation		Usual care
Why			Exercise interventions delivered via telehealth can be safe and effective for improving the quality of life of cancer survivors and offer convenience		Cancer survivors are not routinely offered cancer rehabilitation
What: materials			2× weekly web-based supervised, group-based exercise (approximately 6 participants per group; Zoom) Participants will be provided with an exercise band Participants to receive a web-based (Physitrack), individualized home exercise program, and exercise band Participants to receive access to a web-based information portal (iLearn) with webinars, web-based information handouts, and resources about cancer care and recovery including exercise Participants will wear a physical activity device (Fitbit Inspire) continuously during waking hours for 8 weeks Written information about local exercise services provided at 8 weeks		Standard information booklets about cancer care and recovery including exercise Written information about local exercise services provided at 8 weeks
**What procedures**					
	Provider	One physiotherapist with oncology experience provided by the hospital will provide exercise guidelines in verbal and written format and the web-based group intervention One allied health assistant will support the web-based group intervention		Usual care team (eg, specialist, general practitioner, and allied health professional) One physiotherapist with oncology experience provided by the hospital will provide exercise guidelines in verbal and written format	
	How	Supervised sessions via videoconference supplemented by web-based information above Physical activity device for remote exercise and physical activity monitoring		In person or via telehealth as available	
	Where	Intervention clinicians are clinic-based Participants receive an exercise program at home Participants continue to receive usual cancer care in hospital or at home as indicated		Participants receive usual care in hospital or at home as indicated	
**When and how much**					
	Intensity	Cardiovascular: moderate (BORG 3-5, maximum heart rate=60%-85%) Resistance: 2-3 sets with 10-12 repetitions Participants progressed when completing 3 sets with 10-12 repetitions until fatigue		Advised to undertake physical activity in accordance with current physical activity recommendations: advised to undertake physical activity in accordance with current physical activity recommendations Moderate activity (cardiovascular: maximum heart rate=60%-85%, resistance=2-3 sets, 10-12 repetitions)	
	Frequency	2× weekly supervised 1× weekly unsupervised		Advised to participate in 3x weekly unsupervised physical activity	
	Session time	60-minute web-based exercise group 30-minute unsupervised exercise session		Advised to complete 30-minute unsupervised physical activity	
	Overall duration	8 weeks		8 weeks	
	Tailoring	Individualized exercise program based on initial consultation and goals		None	
	Trial fidelity	Physiotherapists who are employed by the health service to provide the intervention will receive training and mentoring by a senior research physiotherapist with oncology experience Electronic exercise log via the Physitrack app and Fitbit Inspire Records of the content, number, and duration of completed web-based group sessions Clinical supervision of therapists in accordance with standard health service policy Exercise interventions that patients have participated in outside of the trial will be recorded		Physiotherapists who are employed by the health service to provide assessment and advice will receive training and mentoring by a senior research physiotherapist with oncology experience Clinical supervision of therapists in accordance with standard health service policy Exercise interventions that patients have participated in outside of the trial will be recorded	

### Study Outcomes

Participants will complete an assessment of health-related quality of life at weeks 0, 9, and 26. Immediately post intervention (week 9) is the primary end point. Walking capacity and self-efficacy will also be assessed at weeks 0 and 9. Physical activity and health service usage utilization will be assessed at weeks 0 and 26. A clinician blind to group allocation will complete the assessments. Primary and secondary outcomes are outlined in Table 2.

**Table 2:** Primary and secondary outcomes.

Outcomes		Measures or sources	Definitions	Time points
**Primary outcome**				
	Health-related quality of life	European Organisation for Research and Treatment of Cancer Quality of Life Questionnaire	Score on the validated quality-of-life questionnaire before and after the intervention. Primary end point is postintervention.	Week 0 Week 9 Week 26
**Secondary outcomes**				
	Physical activity	activPAL accelerometer	Time spent in moderate to vigorous activity, walking, sitting, and step count before and after the intervention. Participants will wear the activity monitor continuously for 8 consecutive days. Only complete 24-hour recording days will be included for analysis. However, as monitors may need to be removed for the purpose of swimming or bathing, evidence of nonwear matching with an activity logbook will still be included.	Week 0 Week 26
	Physical capacity	6-Minute walk test	Walk distance (in meters) before and after the intervention.	Week 0 Week 9
	Self-efficacy for physical activity	Questionnaire developed using the Health Action Process Approach [34] (Multimedia Appendix 2)	Score on the self-efficacy questionnaire for physical activity before and after the intervention.	Baseline Week 9
	Emergency department presentations	Hospital database and electronic medical record	Number of emergency department presentations during the trial period.	Week 26
	Hospital readmissions	Hospital database and electronic medical record	Number of hospital readmissions during the trial period, associated inpatient days, and duration between with each admission.	Week 26
	Health service utilization	Questionnaire (Multimedia Appendix 3)	Frequency of allied health and medical services, pharmaceutical use, and hospital admissions (external to the health network).	Week 0 Week 26
	Audit of exercise interventions	Questionnaire	Frequency, type, and duration of any exercise interventions completed outside the trial.	Week 9 Week 26

Adverse events as defined by the World Health Organization [35] will be recorded from medical records, direct observation during classes, and by participant self-report to document the safety of the intervention. The event may or may not be related to the intervention but occurs while the person is participating in the intervention phase of the trial. Adverse events will be categorized as either minor or serious and as related or unrelated and expected or unexpected events. A minor adverse event is defined as an incident that occurs while a person is participating in the intervention that results in no injury or minor injury (eg, exacerbation of pre-existing musculoskeletal pain) that requires no or minor medical intervention. A serious adverse event is defined as an incident that occurs while a person is participating in the intervention that results in death, serious injury, or hospitalization (eg, injurious fall resulting in fracture). Serious adverse events will also be recorded for the usual care group. In addition, adverse events will be reported and graded using the Common Terminology Criteria for Adverse Events Version 4 [36]. The consequences of a serious adverse event in the control group (eg, hospitalization and emergency department admission) will be captured by our health service utilization questionnaire and medical record audit. Reasons for nonparticipation in an exercise session or noncompletion of the program will be recorded (eg, pain, fatigue, and unwell).

### Consumer Perceptions

Semistructured interviews will be completed on 2 occasions (immediately after the intervention at week 9 and at week 26) with experimental group participants to explore in detail the experience of people participating in exercise-based cancer telerehabilitation and of behavior change. A purposive sample of participants in the telerehabilitation group will be asked questions relating to satisfaction, barriers, and facilitators to accessing cancer telerehabilitation and perceptions about sustaining physical activity on program completion. The same group will be interviewed at both time points. We will conduct interviews until we reach data saturation; that is, until no new ideas emerge from the data [37]. It is anticipated that this will be achieved by interviewing approximately 20 participants based on our previous qualitative studies of cancer rehabilitation [7,11]. Interviews of approximately 45-minute duration will be conducted in person, via the telephone, or through videoconference, if preferred, by a member of the research team. A different interview schedule at each time point (Multimedia Appendix 4) will be given to participants prior to the interview to allow them to prepare.

Other routinely collected data will be used to describe the sample, including age, gender, cancer type, cancer stage, treatment regimens, comorbidities, baseline functional performance status (AKPS), and baseline BMI.

### Sample Size Estimation

It is estimated that 104 participants will be required to accomplish a power of 0.80 and a 2-tailed α level of .05 to detect a between-group difference of a 10-point change in the EORTC QLQ-C30 score [38] (the minimally important difference established for cancer survivors) assuming an SD of 18 points [39]. Based on our previous trial of cancer rehabilitation [39], we expect a dropout of 10%; therefore, 116 participants will be randomized.

### Statistical Analysis

#### Analysis of Quantitative Data

The primary outcome (postintervention global health–related quality of life) will be analyzed using linear mixed effects models. Modeling will account for variation in baseline values. This method accounts for within-participant dependence of observations over time, and for missing data, allowing some participants to have missing observations at certain time points. If more than 5% of data are missing, a multiple imputation process will be used, providing the assumption that data are missing at random is met. A similar approach will be used for analysis of secondary continuous outcomes collected longitudinally. The time spent in moderate to vigorous physical activity will be estimated using a cut-off of 100 steps per minute for moderate-intensity physical activity [40]. The proportion of participants meeting physical activity guidelines will be described and compared using risk ratios. The number of emergency department and hospital admissions will be reported as an incidence rate ratio using a negative binomial regression model. To avoid bias and to maximize the randomization process, all available data will be analyzed in accordance with allocation (intention-to-treat analysis) regardless of adherence.

Total direct costs to the health service for each participant will be determined from the intervention costs and cost of health services utilized over 6 months as recorded from hospital administrative data and health service utilization questionnaire. Costs associated with delivering telerehabilitation will be attributed to the experimental group and cost associated with usual care will be attributed to the comparison group. These will be determined from a register of staff and the time engaged in telerehabilitation or usual care for each participant. Labor costs will be attributed to the staff member and the cost of the telerehabilitation intervention and usual care (based on time and location) to determine a total intervention cost for each participant as well as infrastructure costs. Total costs for each participant will be determined from the intervention costs, the cost of health services utilized over 6 months for experimental group participants, and the cost of health services utilized over 6 months for comparison group participants. The incremental cost-effectiveness ratio will be calculated as the difference in total program and health service costs per mean difference in the global quality-of-life score between the comparison and experimental groups over 6 months. A cost utility ratio will be calculated on the basis of the EORTC-QLQ C30 global quality-of-life score [41] as the change in total program and health service cost per change in quality-adjusted life years saved in the experimental and comparison groups over 6 months.

#### Analysis of Qualitative Data

Qualitative interview data will be analyzed inductively using interpretive description as a theoretical framework [42]. Interviews will be audio-recorded and transcribed verbatim. Transcripts will be provided to participants to check for accuracy and be given the opportunity to add additional content if they wish. Transcribed interviews will then be deidentified and imported into qualitative analysis software (NVivo [43]). Two researchers will read the interviews and assign codes to sections of the text using an inductive approach, independently. They will then look for connections between and within the data to identify and develop main themes and subthemes using reflective thematic analysis [44]. Once the main themes are decided upon, one researcher will go back and selectively search for text on those themes. Data will be documented using an audit trail including rich and thick descriptions to enhance credibility, trustworthiness, and dependability.

## Results

The trial was funded in April 2021 and registered on October 21, 2021. Participant recruitment commenced in April 2022. As of June 2022, a total of 14 participants were enrolled. Recruitment is expected to conclude in late 2023 and results are expected to be published in 2024.

## Discussion

### Principal Findings

It is hypothesized that patients receiving cancer telerehabilitation will demonstrate improvements in health-related outcomes when compared to usual care without rehabilitation. It is also hypothesized that a cancer telerehabilitation model will be cost-effective and demonstrate high patient satisfaction. These findings will inform future development of cancer rehabilitation programs in hospital-based settings, contributing to the global effort to integrate exercise-based rehabilitation into standard cancer care [1]. Telehealth may be a convenient and effective way to increase access to exercise. However, no previous randomized controlled trials have evaluated supervised, web-based group exercise via videoconferencing in a real-world health setting [15,24]. This trial will compare a comprehensive, exercise-based cancer telerehabilitation program, delivered in a supervised group and usual care on patient and health service outcomes within a pragmatic health service setting.

There are many possible advantages of exercise-based cancer telerehabilitation. Most notable is the possibility to reach a broader population of cancer survivors. Many existing cancer rehabilitation programs are centered in metropolitan areas [3,45]; hence, there is potential to improve access for those in regional and rural areas. Telerehabilitation also may provide an extra element of convenience for a population that usually has a high number of competing medical demands [11]. However, the convenience of telerehabilitation may be countered by the inability to provide exercise interventions with hands-on instruction and the use of specific equipment. There may also be additional challenges related to supervision and exercise monitoring, which may affect the fidelity of telerehabilitation. Despite this possible concern, telerehabilitation generally meets consumer needs [46], and patients have positive views of this type of service delivery [15,16].

### Strengths and Limitations

A strength of this study is the inclusion of health service data. Few studies on cancer rehabilitation include end points meaningful to health services such as hospital length of stay, readmissions, health service utilization, and medication use [47]. This is an issue since costs are a key driver of decision-making in health care. Telerehabilitation has been demonstrated to reduce hospital readmissions compared to usual care in people with advanced-stage cancer and has shown cost savings in other chronic disease settings [48-50]. If shown to be cost-effective, results from this trial may encourage greater implementation of telerehabilitation in cancer settings to improve access to exercise for cancer survivors.

The pragmatic nature of this study implies that a possible limitation is the requirement of participants to have access to their own technology infrastructure to support telehealth. This may bias the population to include participants who have high levels of digital health literacy and access to technology. However, 86% of Australian households have access to the internet, with 91% of them using smartphones and 66% using tablets [51]. This approach is also consistent with the likelihood that future implementation of telerehabilitation programs would be targeted toward people who own suitable devices and have internet access. This trial also does not consider other models of rehabilitation such as 1:1 care. Given that high levels of supervision are important for effectively delivering exercise for cancer survivors [4], possible effects of the telerehabilitation intervention may be diluted owing to the inability to interact 1:1 in a web-based group setting. To account for this, the staff ratio has been kept high to support technology and practical difficulties that patients may encounter.

### Conclusions

Telerehabilitation is a rapidly growing area that may have many positive impacts among cancer survivors. This trial has the potential to inform future models of cancer rehabilitation, which can be implemented in health services to improve access to exercise for cancer survivors.

## Appendix

Multimedia Appendix 1 SPIRIT Table. Schedule of enrollment, interventions, and assessments.

Multimedia Appendix 2 Self efficacy for physical activity questionnaire based on Health Action Process Approach.

Multimedia Appendix 3 Health Service Utilisation questionnaire.

Multimedia Appendix 4 Interview schedule.

## Abbreviations

AKPS: Australian Karnofsky Performance Status
CONSORT: Consolidated Standards of Reporting Trials
EORTC-QLQ C30: European Organisation for Research and Treatment of Cancer Quality of Life Questionnaire

## Data Availability

Data sharing is not applicable to this article as no data sets were generated or analyzed in this study. Data will be made available at the conclusion of the trial in the published multimedia appendices.
