# Supplementary material for: Efficacy of Group Exercise–Based Cancer Rehabilitation Delivered via Telehealth (TeleCaRe): Protocol for a Randomized Controlled Trial
Source: JMIR Res Protoc. 2022 Jul 18;11(7):e38553. doi: 10.2196/38553 (PMC9345024; doi:10.2196/38553)
Supplement: Multimedia Appendix 1 [file resprot_v11i7e38553_app1.doc]

Supplementary File 1. SPIRIT Table. Schedule of enrollment, interventions, and assessments.

|  | **STUDY PERIOD** | | | |
| --- | --- | --- | --- | --- |
|  | **Enrolment** | **Allocation** | **Post-allocation** | |
| **TIMEPOINT** | ***-t_1_*** | **0** | ***t_1_***  ***_Week 9_*** | ***t_2_***  ***_Week 26_*** |
| **ENROLMENT:** |  |  |  |  |
| **Eligibility screen** | X |  |  |  |
| **Informed consent** | X |  |  |  |
| **Allocation** |  | X |  |  |
| **INTERVENTIONS:** |  |  |  |  |
| ***Telerehabilitation*** |  |  |  |  |
| ***Usual Care*** |  |  |  |  |
| **ASSESSMENTS:** |  |  |  |  |
| **HR-QOL** | X |  | X | X |
| **6-minute walk test** | X |  | X |  |
| **Self-efficacy for physical activity** | X |  | X |  |
| **Physical activity** | X |  |  | X |
| **Hospital readmissions** |  |  |  | X |
| **ED presentations** |  |  |  | X |
| **Health service utilisation** | X |  |  | X |
| **Audit of exercise interventions** |  |  | X | X |
| **Participant interviews** |  |  | X | X |

HR-QOL: health-related quality of life; ED: emergency department
