# Supplementary material for: Efficacy of Group Exercise–Based Cancer Rehabilitation Delivered via Telehealth (TeleCaRe): Protocol for a Randomized Controlled Trial
Source: JMIR Res Protoc. 2022 Jul 18;11(7):e38553. doi: 10.2196/38553 (PMC9345024; doi:10.2196/38553)
Supplement: Multimedia Appendix 2 [file resprot_v11i7e38553_app2.docx]

Supplementary File 2. Self efficacy for physical activity questionnaire based on Health Action Process Approach

**Self-efficacy for physical activity**

Please answer the following statement in relation to the circumstances below. Please tick the best answer for you.

Are you confident that you can permanently be regularly physically active?

| ***“I am confident that I can permanently be regularly physically active…”*** | | | | |
| --- | --- | --- | --- | --- |
| … even if I have side-effects (e.g. nausea) of the cancer therapy | Not at all | Partially agree | Mostly agree | Totally agree |
| … even if I am tired | Not at all | Partially agree | Mostly agree | Totally agree |
| … even if I am pain when exercising | Not at all | Partially agree | Mostly agree | Totally agree |
| … even if I am too busy with other activities or appointments | Not at all | Partially agree | Mostly agree | Totally agree |
| … even if I had to exercise alone | Not at all | Partially agree | Mostly agree | Totally agree |
| … even if I felt stressed | Not at all | Partially agree | Mostly agree | Totally agree |
| … even if I felt depressed | Not at all | Partially agree | Mostly agree | Totally agree |
