# Supplementary material for: Efficacy of Group Exercise–Based Cancer Rehabilitation Delivered via Telehealth (TeleCaRe): Protocol for a Randomized Controlled Trial
Source: JMIR Res Protoc. 2022 Jul 18;11(7):e38553. doi: 10.2196/38553 (PMC9345024; doi:10.2196/38553)
Supplement: Multimedia Appendix 3 [file resprot_v11i7e38553_app3.docx]

Supplementary File 3. Health Service Utilisation questionnaire

**Health Service Utilisation Questionnaire at baseline**

Date: _ _/_ _/_ _ Patient Id No. _ _ _ Assessor: _ _ _

**1. Person completing the questionnaire:**

□ Patient □ Other (specify) ________

**2. Current patient location and status: (NB Inclusion criterion – living at home)**

□ At home independent

□ At home with care

□ Other (specify) ___________

**3. Are you currently in paid employment?** □ No □ Yes

**4. If YES to ‘currently in paid employment’ describe type of employment____________**

**5. If YES to ‘currently in paid employment’, have you had to reduce the number of hours per week you work over the last 6 months?** Confirm reason / details below (Tick only one)

□ No, I work the same hours.

□ Yes, I have had to reduce my hours per week by ……………….(hours)

□ Yes, I have had to stop work completely

**6. Are you currently receiving formal home support services?** □ **No** □ **Yes**

**If Yes**, what support have you been receiving in the last 6 months? (tick all that apply)

□ Home help □ Nursing (Eg RDNS)

□ Meals Assistance

□ Other ……………

**8. Healthcare Visits:**

**In the last 6 months, have you needed to visit any of the following health professionals:**

|  | **No** | **Yes** | **Number of times** |
| --- | --- | --- | --- |
| General Practitioner (GP) |  |  |  |
| Medical Specialist |  |  |  |
| Physiotherapist |  |  |  |
| Dietitian |  |  |  |
| Other Allied Health:  *Specify_______________________* |  |  |  |
| Community or District Nurse |  |  |  |
| X-Ray Clinic |  |  |  |
| Pathology Clinic |  |  |  |
| Hospital Emergency Department |  |  |  |
| Hospital Outpatient Clinic |  |  |  |
| Other:  *Specify_______________________* |  |  |  |

**9. In the last 6 months, have you been admitted to a general hospital?**

□ **No** □ **Yes**

**If yes,**

How many times were you admitted to hospital? ___________

What was the total number of nights you spent in hospital? _________nights

In which month(s) were you admitted to hospital? ________________

What was the name of the hospital (s)? _________________________

**10. In the last 6 months, have you been admitted to a rehabilitation hospital?**

□ **No** □ **Yes**

**If yes,**

How many times were you admitted to hospital? ___________

What was the total number of nights you spent in hospital? _________nights

In which month(s) were you admitted to hospital? ________________

What was the name of the hospital (s)? _________________________

**11. Medication use**

List your current prescription medications and how many times you take them each day (OR, please provide a copy of your prescription list):

| **Medication name** | **Dose** | **Number of times per day** |
| --- | --- | --- |
|  |  |  |
|  |  |  |
|  |  |  |
|  |  |  |
|  |  |  |
|  |  |  |

**13. Other comments initiated by the participant:**

Date: _ _/_ _/_ _ Patient Id No. _ _ _ Assessor: _ _ _

**Health Service Utilisation Questionnaire at 6 months**

**1. Person completing the questionnaire:**

□ Patient □ Other (specify) ________

**2. Current patient location and status:**

□ At home independent

□ At home with care

□ Deceased Date / / No further questions (end of questionnaire)

□ Other (specify) ___________

**3. Are you currently receiving formal home support services?** □ **No** □ **Yes**

**If Yes**, what support have you been receiving in the last 6 months? (tick all that apply)

□ Home help □ Nursing (Eg RDNS)

□ Meals Assistance □ Other ……………

**4. Healthcare Visits:**

**Over the last 6 months, have you needed to visit any of the following health professionals other than those at Eastern Health?**

|  | **No** | **Yes** | **Number of times** |
| --- | --- | --- | --- |
| General Practitioner (GP) |  |  |  |
| Medical Specialist |  |  |  |
| Physiotherapist |  |  |  |
| Dietitian |  |  |  |
| Other Allied Health: *Specify___________________* |  |  |  |
| Community or District Nurse |  |  |  |
| X-Ray Clinic |  |  |  |
| Pathology Clinic |  |  |  |
| Hospital Emergency Department |  |  |  |
| Hospital Outpatient Clinic |  |  |  |
| Other: *Specify_______________________* |  |  |  |

**5. During the last 6 months have you been admitted to a general hospital?**

□ **No** □ **Yes**

**If yes,**

How many times were you admitted to hospital? ___________

What was the total number of nights you spent in hospital? _________nights

In which month(s) were you admitted to hospital? ________________

What was the name of the hospital (s)? _________________________

**6. During the last 6 months have you been admitted to a rehabilitation hospital?**

□ **No** □ **Yes**

**If yes,**

How many times were you admitted to hospital? ___________

What was the total number of nights you spent in hospital? _________nights

In which month(s) were you admitted to hospital? ________________

What was the name of the hospital (s)? _________________________

**7. Medication use**

List your current medications and how many times you take them each day (OR, please provide a copy of your prescription list):

| **Medication name** | **Dose** | **Number of times per day** |
| --- | --- | --- |
|  |  |  |
|  |  |  |
|  |  |  |
|  |  |  |
|  |  |  |
|  |  |  |
|  |  |  |

**8. Other comments initiated by the participant:**
