# Supplementary material for: Efficacy of Group Exercise–Based Cancer Rehabilitation Delivered via Telehealth (TeleCaRe): Protocol for a Randomized Controlled Trial
Source: JMIR Res Protoc. 2022 Jul 18;11(7):e38553. doi: 10.2196/38553 (PMC9345024; doi:10.2196/38553)
Supplement: Multimedia Appendix 4 [file resprot_v11i7e38553_app4.docx]

| **Topic Area** | **Sample Questions** |
| --- | --- |
| **Cancer telerehabilitation**  Participants to talk about their experiences and perspectives on the cancer telerehabilitation program | *What was your overall impression of cancer telerehabilitation?*  *What was best about participating in cancer telerehabilitation?*  *What elements of the telerehabilitation program were most useful? (online portal, Fitbits, online group exercise, home exercise program)*  *What elements of the telerehabilitation program were least useful?*  *How could we improve the delivery of cancer telerehabilitation?* |
| **Accessing cancer telerehabilitation**  Participants to discuss how they found accessing the program, including expectations | *Why did you decide to participate in this trial? (prompt: what encouragement from your treating team was provided?)*  *What did you think about participating in an exercise program delivered online before entering the trial? How did your attitude change as the program progressed? (prompts: What telehealth for other appointments have you accessed before? What did you think?)*  *How much effort did it take to organise everything you needed for each session (prompts: was there space, equipment, technology problems)*  *What challenges did you experience with cancer telerehabilitation? How did you overcome these or what would help in the future to overcome these?* |
| **Group exercise via telerehabilitation**  Participants to discuss their feelings about the type of therapy they received | *How ready did you feel to participate in a group exercise class online?*  *How did participating in an online group exercise class affect your ability to be physically active (e.g. motivation, confidence, physical changes)*  *How did you find the exercises provided in class? (prompt: were they the right level for you? Was it flexible/adjusted over time and as needed?)*  *How did participating in group exercise class via video affect therapist/patient interactions?*  *Tell me about how you interacted with other patients participating in the online group exercise class. (prompts: what (if any) support did you provide eachother?)*  *How did this program compare to other exercise experiences you have had (prompt: What advantages/disadvantages were there to delivering exercise online?)Would you recommend telerehabilitation to other people? If so, why? And if not, why not?*  *How likely will you make or sustain changes to your exercise levels now the online exercise program is completed?* |

Supplementary File 4. Interview schedule

**Post-intervention**

**6-month interview schedule (4-months post-intervention completion)**

| **Topic Area** | **Sample Questions** |
| --- | --- |
| **Behaviour change** | *Tell me about your current exercise levels.*  *How have you applied the information from the cancer telerehabilitation program to change your exercise levels since you completed the program?*  *What challenges have you experienced in being physically active since completing the program?*  *What has helped you maintain your physical activity since participating in the program?* |
| **Attributing change** | *What motivates you to exercise? (e.g. family support, online resources, written instructions, access to gym/supervision, feeling well etc.)*  *How do you think telerehabilitation contributed to your current exercise levels?* |
| **Intention of future change** | *What changes, if any would you like to make in the next 6 months to your exercise habits?*  *What support do you need to make changes to your future exercise habits?* |
